# Integrating experimental and network pharmacology to explore the pharmacological mechanisms of Dioscin against glioblastoma

**DOI:** 10.1515/med-2025-1194

**Published:** 2025-05-13

**Authors:** Jianhui Huang, Caiyun Jiang, Tingting Li, Zhongdong Hu, Qiaoling Xiang, Hongxia Chen

**Affiliations:** Department of Pharmacy, The Sixth Affiliated Hospital of Jinan University, Dongguan, 523576, China; Department of Pharmacy, The Third Affiliated Hospital of Sun Yat-sen University, Guangzhou, 510630, China; Laboratory Animal Center, Peking University Shenzhen Graduate School, Shenzhen, 518055, China

**Keywords:** Dioscin, glioblastoma, network pharmacology, apoptotic pathway, EGFR

## Abstract

**Background:**

Dioscin (Dio) is an important anti-tumor active component found in the seeds of *Livistona chinensis*. However, the efficacy and mechanism of Dio in relation to the progression of glioblastoma (GBM) remain unclear.

**Materials and methods:**

Using 3-(4,5-Dimethylthiazol-2-yl)-2,5-diphenyl-2H-tetrazolium bromide assays, flow cytometry, and Hoechst staining *in vitro* experiments. A tumor-bearing nude mouse model was employed to further explore the impact of Dio on GBM tumor growth *in vivo* experiments. Using network pharmacological analysis and molecular docking to predict the potential target proteins and signaling pathways of Dio anti-GBM. The expression of proteins associated with apoptotic pathways was assessed by western blotting.

**Results:**

Dio effectively suppresses the proliferation and promotes apoptosis of U251 cells. In the established tumor-bearing nude mouse model, the anti-cancer activity of Dio was further assessed. Kyoto Encyclopedia of Genes and Genomes enrichment analysis highlighted cancer signaling pathways, including epidermal growth factor receptor (EGFR). Western blot results showed that EGFR phosphorylation and apoptosis gene CASP3 increased with the increase of Dio concentration.

**Conclusions:**

Dio could inhibit the proliferation and promote apoptosis of GBM cells, and play a significant inhibitory role in tumor growth. Dio could affect the phosphorylation of EGFR and then trigger the apoptosis process, resulting in the up-regulation of apoptosis protein CASP3 expression in GBM cells.

## 1 Introduction

Chinese medicine has increasingly been utilized in the treatment of malignant tumors. *Livistona chinensis*, a plant from the Palmaceae family, has dried fruit widely used in traditional practices and possesses reliable pharmacological activity. Dioscin (Dio), an important anti-tumor component found in the seeds of *L. chinensis*, influences various stages of tumorigenesis and development [1–4]. A number of studies have shown that Dio can play an anti-tumor role by inducing tumor cell cycle arrest, DNA damage, activating mitochondrial signaling pathway, and inhibiting epithelial–mesenchymal transformation of tumor cells [5–7]. Chae and Kim found that Dio significantly inhibits the proliferation of breast cancer cells [8]. *In vitro* studies have shown that Dio induces apoptosis and inhibits proliferation in prostate cancer cells [9] and promotes apoptosis in oral squamous cell carcinoma (OSCC) cells [10]. Chen et al. found that Dio can inhibit TGF-β1-induced EMT of liver cancer cells, reduce the invasion and migration of liver cancer cells, reduce the activity of TGF-β1-induced signaling pathways, and play an anti-liver cancer role [11]. Overall, Dio plays an important role in promoting apoptosis and inhibiting proliferation of cancer cells in a variety of cancers.

Glioblastoma (GBM) is the primary malignancy of the central nervous system and is highly aggressive and heterogeneous, has a high recurrence rate, poor prognosis, a median survival time of only 15 months, and a 5-year survival rate of <10% [12–14]. The main treatments for gliomas include surgery [15], radiotherapy, and chemotherapy [16,17]. Temozolomide (TMZ) is the most extensively studied chemotherapeutic agent, demonstrating clear efficacy in GBM treatment [18]. However, the overall survival rate for GBM has not significantly improved, and both clinical prevention and treatment continue to pose major challenges [19]. For example, the blood–brain barrier (BBB) is difficult to break through, the efficacy of therapies developed partly based on immune cells is not significant, the heterogeneity of tumor cells is strong, the immunosuppression of tumor microenvironment, and the adverse reactions of drugs themselves, so that the overall prognosis of GBM patients is still poor. Therefore, exploring novel and effective therapies for GBM is essential. Currently, there are no detailed reports on Dio’s influence on GBM, which makes our in-depth study on Dio’s anti-GBM more innovative and valuable.

Network pharmacology is an emerging discipline based on the theories of systems biology, genomics, proteomics, and other omics disciplines. It uses high-throughput omics data analysis, computer simulation, and open database retrieval techniques to reveal the network relationship of drug gene–target–disease interactions. It has become a key method to study pharmacological mechanisms at the molecular level [20–23].

**Figure 1:**
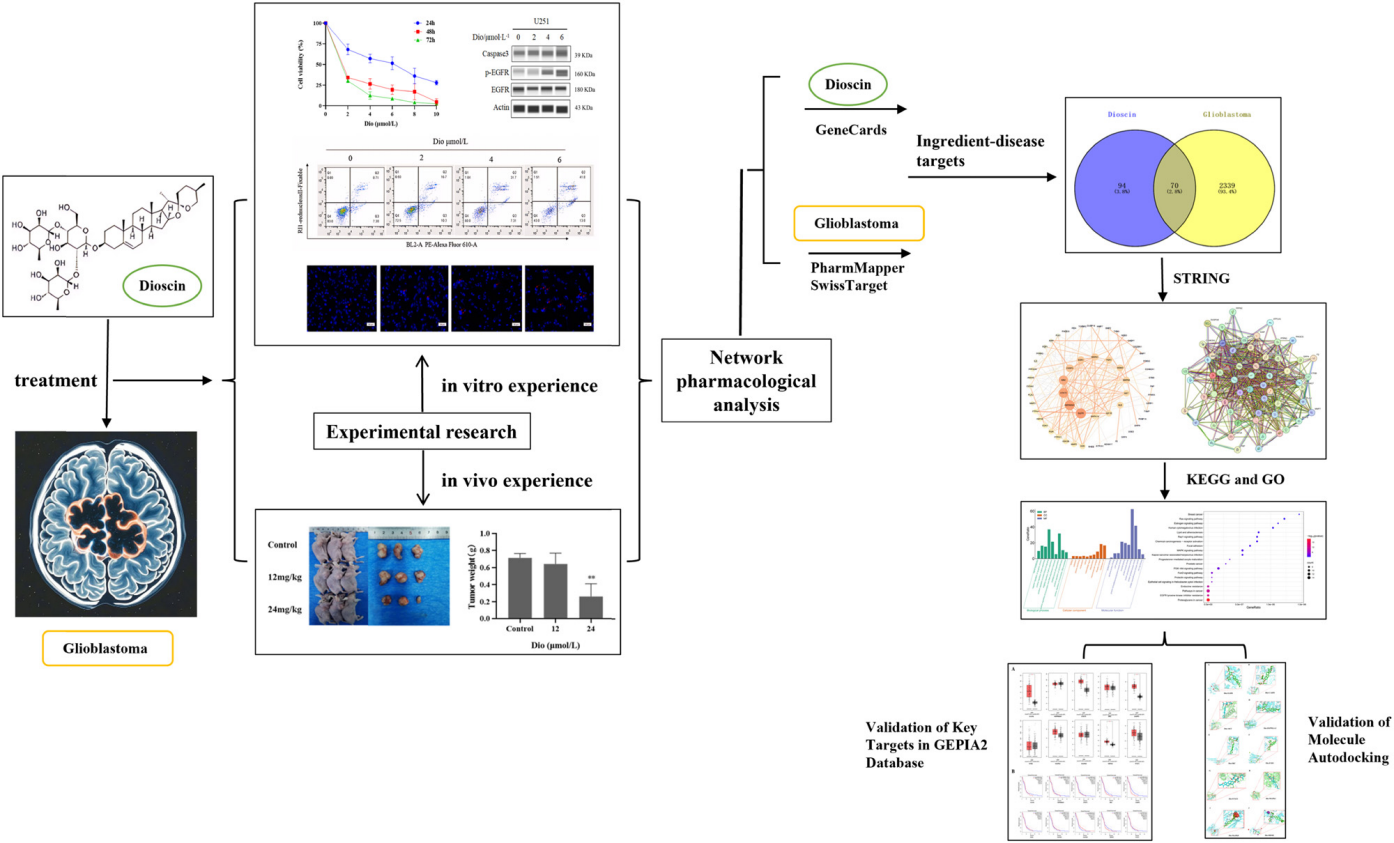
Flow chart of this study.

Our research focus is to use network pharmacology to further analyze the possible pharmacological mechanism of Dio on GBM on the basis of *in vivo* and *in vitro* experimental studies. *In vitro* and *in vivo* experiments, we found that Dio could effectively inhibit the proliferation of GBM cells, promote cell apoptosis, and inhibit the growth of tumors in tumor-bearing nude mice. More importantly, we integrated multiple database resources to conduct a multi-level analysis of the interaction between Dio and GBM using network pharmacological methods, and screened the core targets of Dio and GBM interaction. We found that the core target proteins of Dio–GBM interaction include epidermal growth factor receptor (EGFR), which may be closely related to apoptosis signaling pathway. The primary verification of the core target was conducted by western blotting experiments, and it was found that EGFR phosphorylation and apoptosis gene CASP3 increased with the increase of Dio drug concentration. This may suggest that the mechanism by which Dio acts on GBM cells is to affect the phosphorylation of EGFR, then regulate its downstream signal transduction, and finally trigger the apoptosis process, resulting in the up-regulation of CASP3 expression, a key gene for apoptosis. This study provides a new idea for the treatment of GBM and facilitates the clinical translation of Dio therapy for GBM. The methodology of this research is summarized in the flowchart (Figure 1).

## 2 Materials and methods

### 2.1 Cell culture and Dio treatment

U251 cells (Guangzhou Women and Children Medical Center, Guangdong Province, China) were cultured in Dulbecco’s modified eagle medium (DMEM) supplemented with 10% fetal bovine serum (FBS) in 10 mm cell culture dishes at 37°C and 5% CO_2_. The cells were treated with Dio (D114066, Shanghai Aladdin Biochemical Technology Co., Ltd, Shanghai, China) at varying concentrations (0–10 μM) in DMEM containing 10% FBS and incubated for 24 h or longer. All experiments were performed at the Laboratory Animal Center of Peking University Shenzhen Graduate School.

### 2.2 3-(4,5-Dimethylthiazol-2-yl)-2,5-diphenyl-2H-tetrazolium bromide (MTT) assay

U251 cells were cultured in 96-well plates until reaching 80% confluency and then treated with different concentrations of Dio (0, 2, 4, 6, 8, and 10 μM). After incubation at 37°C and 5% CO_2_ for 24 h, 10 μL of MTT solution (Shanghai Yingxin Laboratory Equipment Ltd, Shanghai, China) was added to each well, followed by an additional 6 h incubation. The supernatant was then removed, and 150 μL of dimethyl sulfoxide (DMSO) was added to each well. Absorbance values were measured at OD570 nm using a microplate reader (Labserv K3 Thermo Fisher Scientific Inc., USA). U251 cells were also treated for 48 and 72 h to assess cell viability.

### 2.3 Cells apoptosis assay

U251 cells were treated with Dio at different concentrations (0, 2, 4, and 6 μM) for 24 h, then collected and stained using the Annexin V-PE/Red Nucleus II Apoptosis Detection Kit (UELANDY Biotechnology Co., Suzhou, China) according to the manufacturer’s instructions. Apoptotic cells were examined using FACSort flow cytometry (Invitrogen, USA), and the apoptosis data were analyzed with FlowJo software.

### 2.4 Hoechst staining assay

U251 cells were cultured in 6-well plates until reaching 80% confluency and then incubated with or without Dio for 24 h. Following this, 4% formaldehyde was added to the cells for 24 h at 4°C, and they were subsequently stained with Hoechst 33342 (Cat. No. C0031) for 30 min. Finally, the cell staining was observed using a fluorescence microscope (OLYMPUS, Japan).

### 2.5 Animal experiment

Nine 4-week-old BALB/c nude mice (18 ± 20 g) were obtained from Beijing Vital River Laboratory Animal Technology Co., Ltd (Beijing, China). The mice were housed under controlled conditions (19–23°C) with a 12 h light/dark cycle and provided standard food and water. After acclimation for 1 week, the mice were randomly divided into three groups, each containing three mice. Xenograft models were established by subcutaneously inoculating human U251 cells (2 × 10^6^ cells in 200 μL phosphate-buffered saline [PBS]) into the right flank of the nude mice. Dio was dissolved in a vehicle solution containing 1% DMSO (Sigma-Aldrich) in PBS. The solution was freshly prepared daily and sonicated for 5 min at 37°C to ensure complete dissolution. Six mice received intraperitoneal injections of Dio at doses of 12 and 24 mg/kg (administered in a volume of 10 mL/kg body weight) daily for 28 days, while the remaining three served as the control group received an equivalent volume of vehicle solution (1% DMSO in PBS) without Dio treatment. During the treatment period, animal body weight and behavioral status (including activity level, feeding, and signs of distress) were recorded every 3 days to assess drug tolerance. No significant weight loss (>10%) or adverse effects were observed in any group. After 4 weeks of treatment or when xenografted tumors reached a diameter of 1.5 cm, the mice were euthanized using excess carbon dioxide gas. For the euthanasia, all the mice were placed in a sealed container, employing a controlled and precise approach, CO_2_ is introduced into a 10 L hermetically sealed container at a rate ranging from 1 to 3 L/min, until their breathing and heartbeats ceased. Tumor tissue was then excised and weighed.

### 2.6 Western blotting

Protein was extracted from cultured U251 cells using RIPA lysis buffer (P0013B, Beyotime, Shanghai, China). The total protein concentration was determined using a BCA kit (P0010, Beyotime, Shanghai, China) according to the manufacturer’s instructions. Protein isolation and detection were performed using an automated capillary electrophoresis system (Simple Western system and Compass software; ProteinSimple) [24]. The following antibodies were used: anti-Caspase 3 (664702-2-lg, Proteintech, 1:50), anti-phospho EGFR (Tyr1069) (30277-1-AP, Proteintech, 1:10), EGFR (66455-1-lg, Proteintech, 1:250), and anti-β-actin (66009-1-lg, Proteintech, 1:5,000). Results were visualized using ProteinSimple software.

### 2.7 Acquisition of potential targets for Dio and GBM

Using the Pharmmapper database (http://www.lilab-ecust.cn/pharmmapper/), we predicted target proteins associated with Dio and selected the top 150 targets based on a median Norm Fit ≥ 0.58. These targets were standardized using UniProt (https://www.uniprot.org/) and the NCBI Gene module. Additionally, we employed the SwissTarget Prediction database (http://www.swisstargetprediction.ch/) with a filtering condition of >0.1 to identify further Dio-related targets, yielding a total of 18 additional targets. By combining the targets from both databases and removing duplicates, we ultimately identified 164 target proteins associated with Dio. GBM-related disease targets were retrieved from the GeneCards database (https://www.genecards.org/). A total of 1,897 GBM-related targets and 164 Dio-related targets were selected for intersection analysis, which was visually represented using a Venn diagram. The overlapping targets were considered as Dio targets associated with GBM.

### 2.8 Construction of protein–protein interaction (PPI) network and screening of core targets

Cytoscape 3.6.0 software was used to analyze the target network of GBM, and the intersection of Dio targets for GBM was mapped into a PPI network using the STRING database (https://cn.string-db.org), selecting “Homo sapiens” and applying a confidence score filter of ≥0.7.

### 2.9 Gene ontology (GO) functional enrichment analysis and Kyoto Encyclopedia of Genes and Genomes (KEGG) pathway enrichment analysis

Furthermore, GO function and KEGG pathway enrichment analyses (https://www.bioinformatics.com.cn/) were performed on the core network targets of the PPI network to screen Dio targets for GBM.

### 2.10 Molecular docking

The analysis of molecular docking is used to predict ligand–receptor target proteins interactions. We used the RCSB PDB database (http://www.rcsb.org/) and downloaded the 3D structures of ten targets proteins. Dehydrating the three receptor proteins using PyMOL software, and then hydrogenated and charged these receptor proteins using Autodock software. Meanwhile, we downloaded the 2D structure of ligand compounds from the PubChem database (https://pubchem.ncbi.nlm.nih.gov), followed by energy minimization processing and data export using ChemBio3D software. Subsequently, Autodock Vina (http://vina.scripps.edu/) software was used to dock the treated ligands with the receptor proteins in order to obtain free binding energy analyzed by the software. Finally, we visualized the results using PyMol software.

### 2.11 Validation of key targets in GEPIA2 database

The online tool of GEPIA2 (http://gepia2.cancer-pku.cn/index.html) was used to verify the expressions of ten key targets in TCGA.

### 2.12 Statistical analysis

The data were analyzed using one-way analysis of variance and are presented as mean ± SD. Statistical analysis and graph creation were performed using GraphPad Prism 8.0 software. Statistical significance was defined as *P* < 0.05.


**Ethical approval:** This animal research was approved by the Animal Ethics Committee of the Laboratory Animal Center of Peking University Shenzhen Graduate School (AP number: AP0044003).

## 3 Results

### 3.1 Effect of Dio on proliferation and apoptosis key pathway of U251 cells

**Figure 2:**
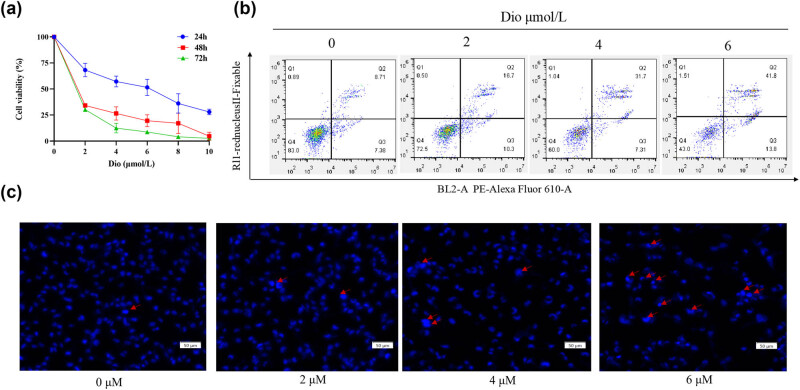
CCK8, flow cytometry, and Hoechst staining assay. (a) Effects of Dio on the proliferation inhibition rate of U251 cells. (b) Effect of Dio on the apoptosis of U251 cells. (c) After Hoechst dye staining, the apoptosis of U251 cells treated with Dio was observed by fluorescence microscope and identified by red arrows. The magnification was 10×.

Compared with the control group, different concentrations of Dio treated U251 cells for 24, 48, and 72 h the cell proliferation inhibition rate was significantly increased, and showed a certain concentration–time dependence in Figure 2a. The half-inhibitory concentration (IC_50_) of Dio on U251 cells at 24, 48, and 72 h, respectively, are 4.931, 1.172, and 1.061.

Through flow cytometry analysis, the apoptosis rate of U251 cells treated with different concentrations of Dio was significantly increased compared with the control group, as shown in Figure 2b. In addition, Hoechst 33342 was used to stain U251 cells treated with different Dio concentrations, and the results showed that the number of cell nuclei with concentrated or fragmented apoptosis in each treatment group was significantly higher than that in the control group (Figure 2c).

### 3.2 Dio inhibited tumor growth *in vivo*

**Figure 3:**
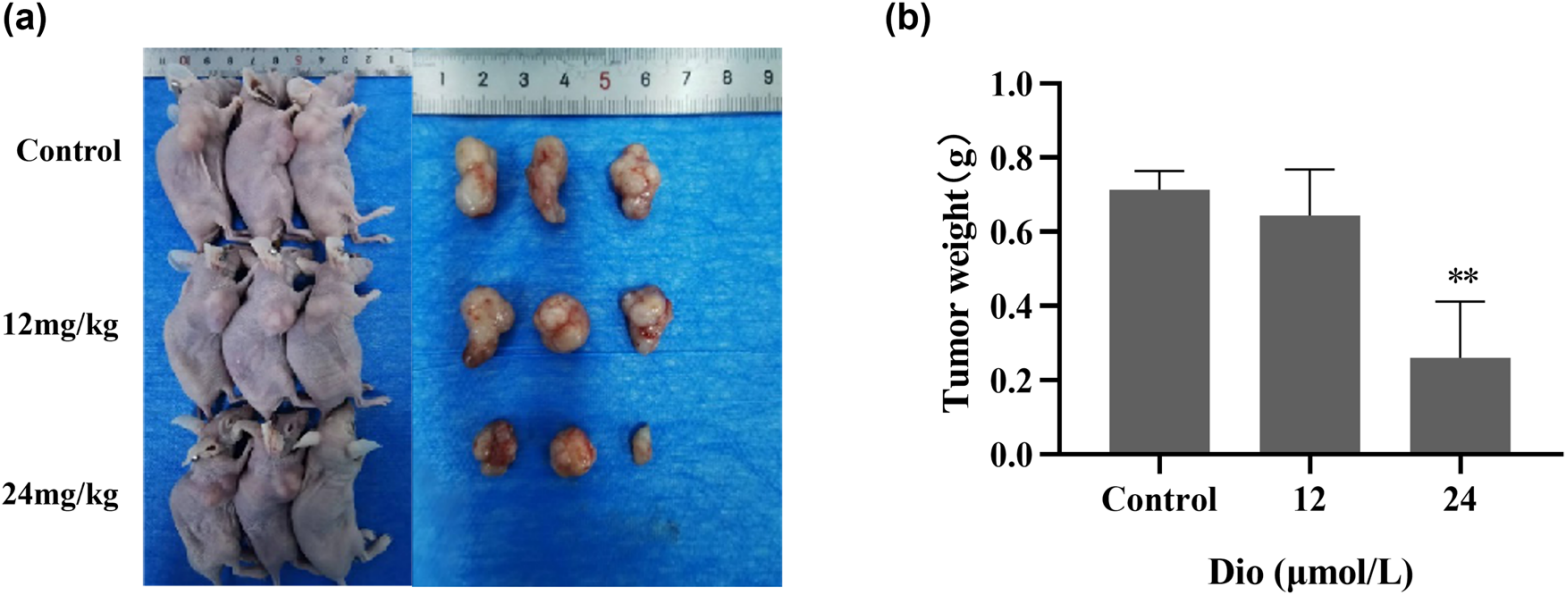
Dio inhibited tumor growth *in vivo*. (a) Tumor growth was monitored by bioluminescence imaging technique, and the tumor growth size and subcutaneous xenograft tumor tissue were photographed. Shown are representative images from at least three independent experiments. (b) Tumor weight was monitored as described in (a). **P ＜ 0.01.

Then, we further investigated the effect of Dio treatment on tumor-bearing nude mice model which was constructed by subcutaneously injecting U251 cells. PBS or Dio were injected into tumor-bearing nude mice model intraperitoneally, and tumor growth was examined. As expected, tumors formed by Dio treatment grew much slower than that formed by PBS after 28 days (Figure 3a). The mice were euthanized and xenograft tumor tissue was dissected. As can be seen from Figure 3a and b, the tumor volume and weight of Dio-treated mice were significantly reduced compared with those that were not treated with Dio.

### 3.3 Dio targets for GBM

**Figure 4:**
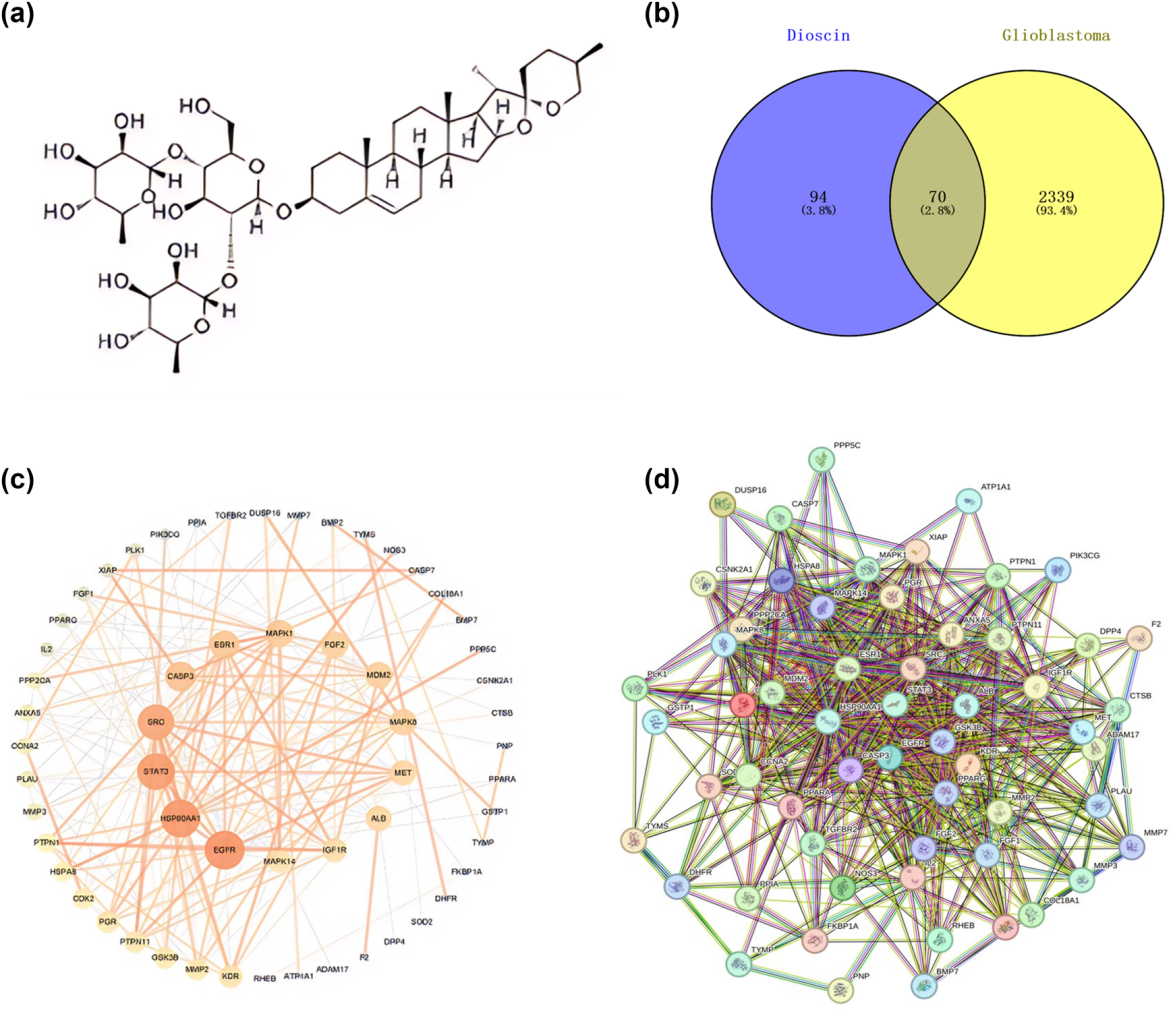
Screening for Dio-related targets and PPI network analysis. (a) Chemical structure of Dio. (b) Venn diagram illustrating the overlap of Dio targets and GBM disease targets. (c) Yellow nodes represent targets with higher degree values. (d) Visualization of interactions among overlapping genes.

We screened a total of 1,897 GBM targets using the Genecards and Disgenet databases. Subsequently, 164 genes related to Dio (chemical structure shown in Figure 4a) were identified through SwissTarget Prediction and Pharmmapper databases. Venn diagram analysis was then performed to determine the intersection targets, resulting in the identification of 70 specific Dio targets for GBM (Figure 4b).

### 3.4 PPI network analysis and core target acquisition

The interaction relationships among the 70 intersection targets were analyzed using the STRING database, and the results were visualized with Cytoscape software (Figure 4c and d). The degree values, indicating core targets with the highest connectivity, were calculated through network analysis. The top ten targets based on these values are listed in Table 1.

**Table 1:** Core target information

No	Targets name	Uniplot ID	Degree
1	EGFR	EGFR_HUMAN	24
2	HSP90AA1	P07900	23
3	STAT3	P40763	22
4	SRC	SRC_HUMAN	21
5	CASP3	P42574	16
6	ESR1	ESR1_HUMAN	15
7	MAPK1	MK01_HUMAN	14
8	MAPK8	MK08_HUMAN	13
9	MDM2	MDM2_HUMAN	13
10	FGF2	P09038	13

### 3.5 GO and KEGG analysis of Dio targets for GBM

**Figure 5:**
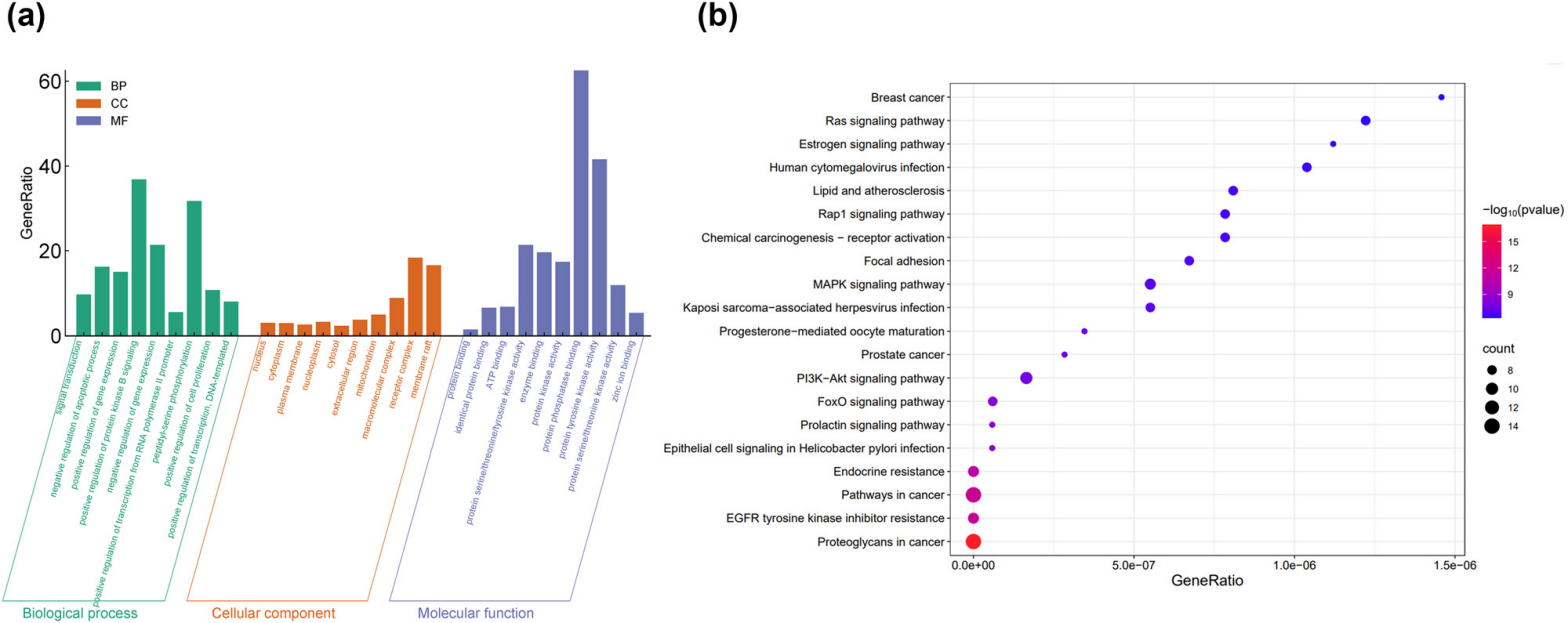
GO and KEGG analysis: (a) enrichment analysis of potential targets of Dio treatment of GBM and (b) KEGG pathway.

GO enrichment analysis identified the top ten core targets (Figure 5a). The KEGG pathway analysis is presented in Figure 5b, highlighting that Dio primarily impacts pathways related to (1) EGFR tyrosine kinase inhibitor (TKI) resistance, (2) pathways in cancer, (3) PI3K-Akt signaling pathway, and (4) proteoglycans in cancer. We selected the EGFR pathway, because it is linked to apoptosis, for further investigation.

### 3.6 Interactions of top ten protein targets and Dio

**Figure 6:**
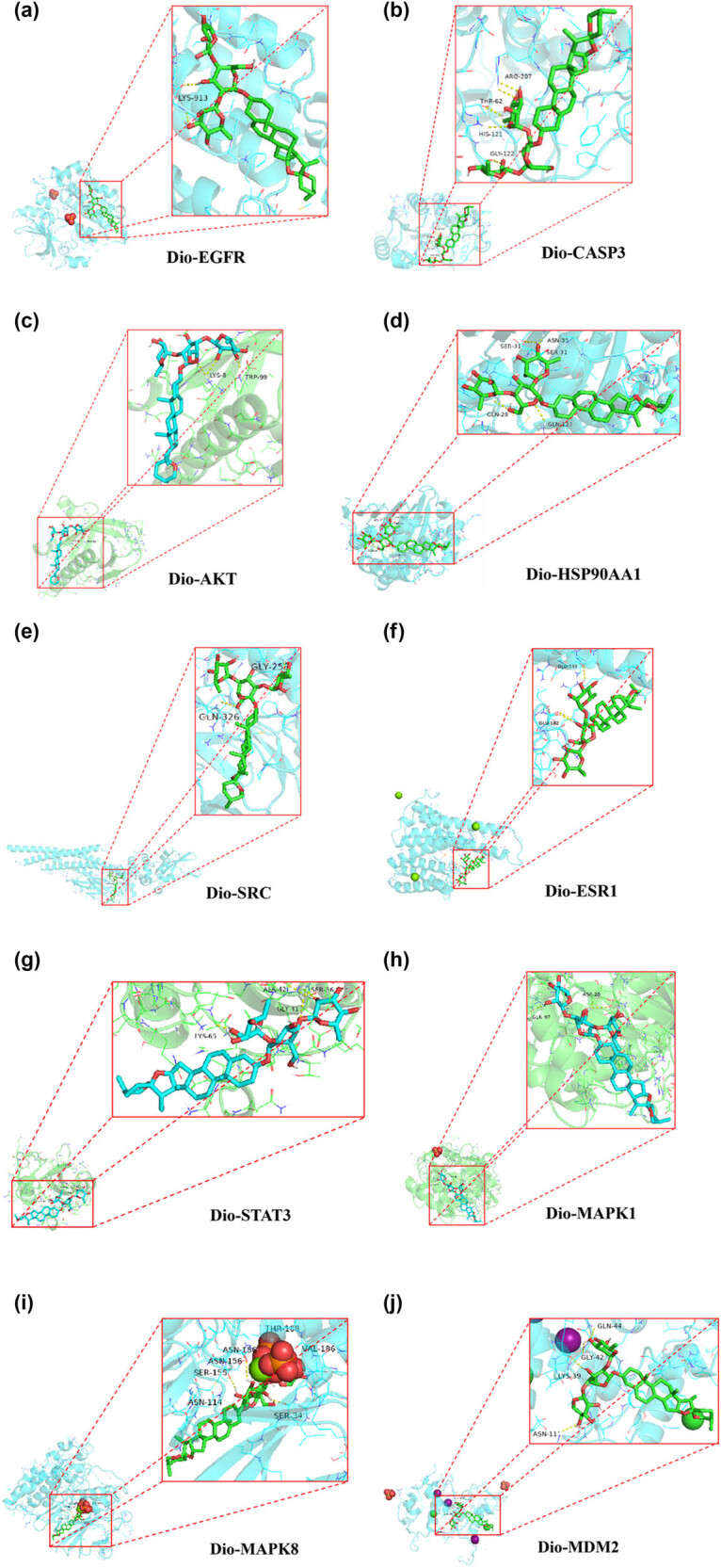
Molecular docking map of active ingredients with key targets: (a) Dio-EGFR, (b) Dio-CASP3, (c) Dio-AKT, (d) Dio-HSP90AA1, (e) Dio-SRC, (f) Dio-ESR1, (g) Dio-STAT3, (h) Dio-MAPK1, (i) Dio-MAPK8, and (j) Dio-MDM2.

Dio was subjected to molecular docking with ten core targets. A lower binding energy between the ligand and the receptor indicates a more stable structure and a higher likelihood of biological activity. When adhering to a docking energy constraint standard below 7.8, the results showed that Dio exhibits strong binding affinity to multiple core targets (Table 2), the corresponding docking model is illustrated in Figure 6.

**Table 2:** Results of molecular docking

No	Target name	PDB ID	Energy (kcal/mol)
1	EGFR	1xkk	−9.0
2	CASP3	1nme	−7.9
3	AKT	1unq	−7.8
4	HSP90AA1	5j2x	−9.1
5	SRC	1o43	−8.2
6	ESR1	8bzw	−9.0
7	STAT3	6njs	−9.3
8	MAPK1	8aof	−9.0
9	MAPK8	4qtd	−9.9
10	MDM2	5c5a	−9.7

### 3.7 Validation of the key targets in GEPIA2 database

**Figure 7:**
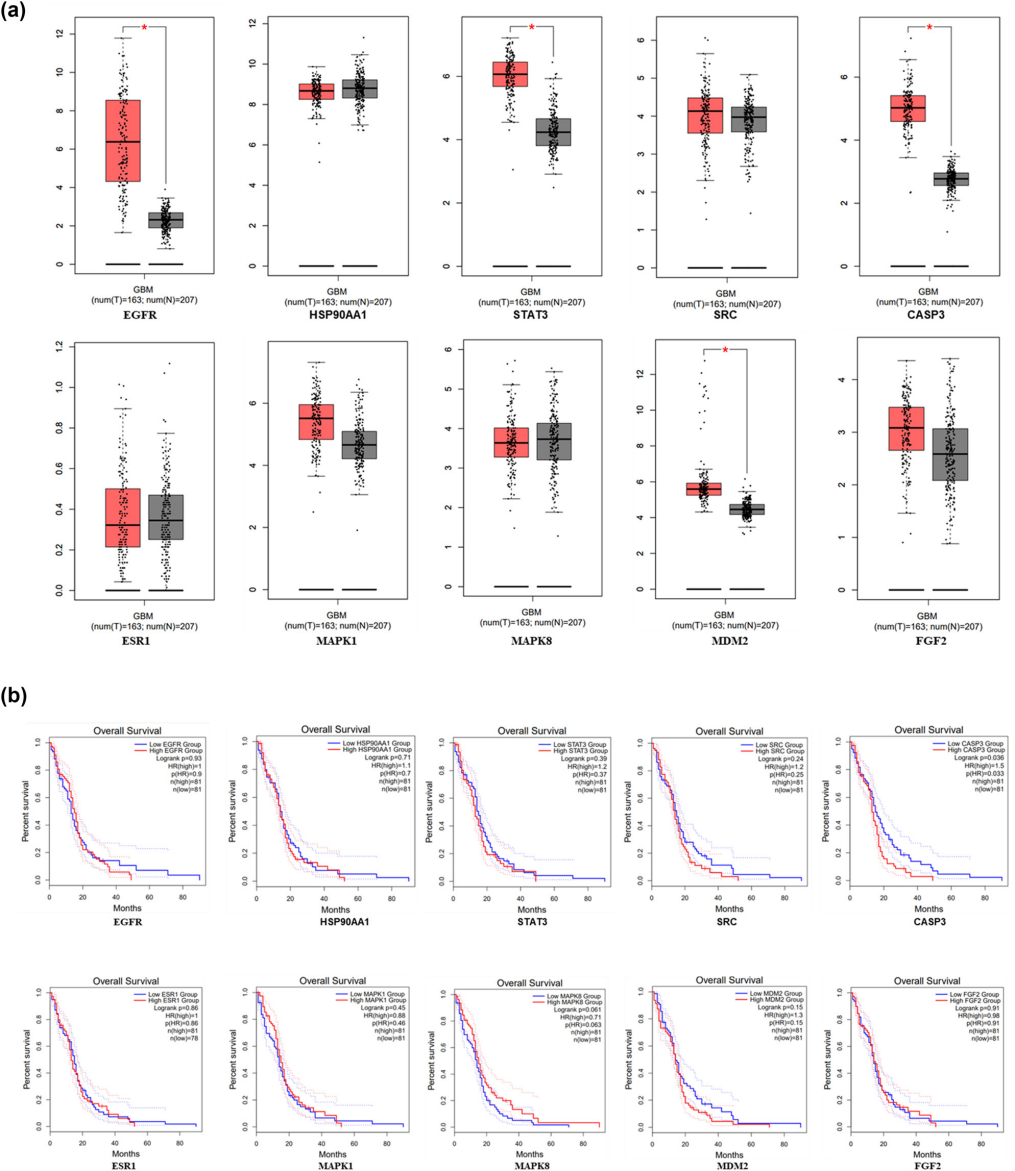
Expression and overall survival of hub genes in GEPIA2 database. (a) Expression of hub genes in GEPIA2 database, the red box plots represent tumor and the gray box plots represent normal. (b) Overall survival of hub genes in GEPIA2 database, the blue represents low expressions and the red represents high expression.

The expressions of hub genes in the GBM samples were investigated in GEPIA2 database. The results showed that mRNA expressions of EGFR, STAT3, CASP3, and MDM2 were significantly higher in GBM tissues than in normal tissues (*P* < 0.05) (Figure 7a). The overall survival was searched in GEPIA2 database. The results indicated that the high expressions of CASP3 were associated with a significantly poor survival (*P* < 0.05) (Figure 7b).

### 3.8 Effect of Dio on apoptosis key pathway of U251 cells

**Figure 8:**
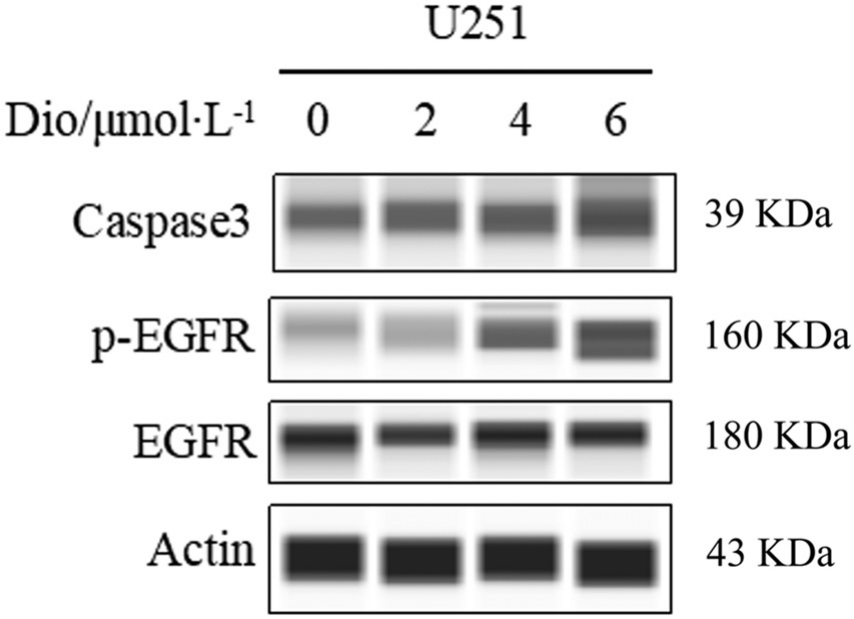
Western blotting analysis of Caspase3, p-EGFR protein, and EGFR after Dio (0, 2, 4, 6 μM) treatment in U251 cells for 24 h.

Western blot results showed that following treatment with Dio, the apoptosis protein expression of Caspase3 was clearly increased which was consistent with flow cytometry results. Moreover, elevated p-*EGFR* was observed in U251 cells after Dio (0, 2, 4, 6 μM) treatment for 24 h (Figure 8).

## 4 Discussion

Malignant GBM is a highly aggressive cancer characterized by a poor prognosis due to the challenges of complete surgical resection and resistance to radiation and chemotherapy [25]. The standard treatment involves surgical resection, followed by chemotherapy with TMZ and postoperative radiotherapy [26]. However, a curative treatment remains elusive, highlighting the urgent need for effective therapeutic agents to treat GBM and prevent tumor recurrence. Chemoprevention has emerged as a promising strategy for managing GBM [27,28]. Dio, a steroidal saponin derived from the seeds of *L. chinensis*, has shown significant antitumor properties against various cancers [8,29–33]. Despite its potential, the preventive effects of Dio on GBM and its underlying mechanisms remain largely unexplored.

This study found that Dio is an effective antitumor drug, which can play an anti-GBM role both *in vivo* and *in vitro*. *In vitro* experiment results showed that Dio could inhibit the proliferation of U251 cells and effectively promote the apoptosis of U251 cells. To evaluate the antitumor effect of Dio *in vivo*, we established a tumor-bearing nude mouse model. *In vivo* studies have shown that Dio can effectively inhibit tumor growth induced by subcutaneous U251 cells in tumor-bearing nude mice, suggesting that Dio has the potential to be used as a clinical transforming drug against GBM, and stimulating our interest in further exploring the pharmacological mechanism of Dio against GBM.

Following this, we identified ten potential core targets where Dio might play a role in GBM prevention through network pharmacological analysis, particularly two key apoptosis-related targets, EGFR and CASP3. CASP3, a member of the cysteine protease family, plays a key role in mediating neuronal apoptosis [34]. Under pathological conditions, BCL-2 family members on the damaged mitochondrial membrane mediate the release of cytochrome c into the cytoplasm, inducing the cleavage of CASP3 and activating it into cracked CASP3, thus initiating the apoptosis process [35,36]. According to literature reports, the malignancy degree and prognosis of lymphoepithelial tissue can be determined by the level of CASP3, which also plays a certain role in the diagnosis and treatment of lung cancer, and its expression is up-regulated in invasive lung cancer cells [37]. Other studies have shown that AKT overexpression can inhibit CASP3 activation and promote tumor progression [38]. PI3K/AKT pathway inhibitors combined with EGFR-targeting drugs have been proposed for the treatment of head and neck squamous cell carcinoma [39]. In addition, the regulatory role of EGFR-AKT on the signaling axis has been shown to inhibit pancreatic cancer progression [40].

Studies have demonstrated that Dio exerts multi-target regulatory effects on the EGFR signaling pathway and its downstream effectors. In OSCC, Dio suppresses the binding of EGFR to the AT-rich sequences in the survivin promoter, significantly downregulating survivin expression and thereby inducing tumor cell apoptosis [41]. In EGFR-mutated lung adenocarcinoma models with TKI resistance, Dio transcriptionally inhibits the expression of tyrosine phosphatase SHP2 through reactive oxygen species (ROS)-dependent activation of p53. This disruption of SHP2–GAB1 interaction ultimately leads to dual suppression of the MEK/ERK and PI3K/AKT signaling pathways [42]. Such mechanisms are critical in overcoming TKI resistance, suggesting that Dio inhibits pro-survival signaling by targeting the EGFR-MAPK/PI3K-AKT axis. Furthermore, network pharmacology analyses reveal that Dio and its metabolite, diosgenin, directly bind to tyrosine kinase receptors such as EGFR, interfering with phosphorylation activation and subsequently inhibiting the Ras-MAPK and PI3K-Akt pathways [43]. Notably, while Dio upregulates EGFR expression via the Notch1/Jagged1 pathway in liver regeneration models [44], it exhibits suppressive effects on EGFR activity in tumor contexts. However, this study in GBM models revealed that Dio treatment induced a significant concentration-dependent upregulation of EGFR phosphorylation levels, accompanied by concurrent activation of the apoptotic marker CASP3. This phenomenon suggests that Dio’s regulation of EGFR signaling may exhibit tumor type or microenvironment-dependent characteristics. Notably, studies have shown that EGFR in GBM frequently exhibits gene amplification (∼50% of cases) or mutations (e.g., EGFRvIII), leading to constitutive activation of its signaling pathway with distinct regulatory mechanisms compared to other tumors [45]. Dio might exert differential effects by targeting abnormal EGFR conformations (such as EGFRvIII) or compensatory signaling feedback mechanisms specific to GBM. Furthermore, research indicates that the EGFRvIII mutant lacks a ligand-binding domain and exhibits ligand-independent signaling activation [46], which could potentially alter Dio’s molecular targets or downstream effects. In summary, the unique genetic landscape of GBM (e.g., high-frequency EGFR amplification/mutation) and its immunosuppressive microenvironment may fundamentally reshape Dio’s mechanism of action. The observed EGFR phosphorylation might activate pro-apoptotic branches (e.g., JNK/p38 pathway) rather than classical pro-survival pathways (PI3K/AKT). The early activation of CASP3 could potentially cleave EGFR to generate pro-apoptotic truncated fragments (p60 EGFR), establishing a positive feedback loop for apoptosis. In this regard, our future research can focus on (1) elucidating Dio’s specific regulation of different EGFR phosphorylation sites, (2) comparing Dio’s effects in EGFR wild-type vs EGFRvIII mutant GBM models, and (3) deciphering the causal relationship between EGFR phosphorylation upregulation and downstream pro-apoptotic signaling. This study first unveils a non-classical mechanism through which Dio induces apoptosis via the EGFR–CASP3 axis in gliomas, providing novel insights for developing therapeutics targeting EGFR signaling networks.

Given that apoptosis-associated proteins are involved in multiple cancer signaling pathways, we combined with network pharmacological analyses to speculate that Dio may interact with EGFR and CASP3 in GBM. Through molecular docking and verification of key targets in the GEPIA2 database, we found that Dio had a strong binding affinity for the core targets of EGFR and CASP3, and the expression of EGFR and CASP3 mRNA in GBM tissues was significantly higher than that in normal tissues, and high expression of CASP3 was associated with a significantly lower survival rate. The verification results were consistent with the results of network pharmacological analysis. Interestingly, in the experimental study of GBM U251 cells treated by Dio, western blotting results showed that the EGFR phosphorylation and apoptosis gene CASP3 increased with the increase of Dio drug concentration. We hypothesize that increased EGFR phosphorylation may activate a series of downstream signaling pathways. In GBM, the abnormally activated EGFR signaling pathway is associated with a variety of malignant biological behaviors of tumor cells, and the elevation of CASP3 indicates the beginning of apoptosis process. This may imply that the mechanism by which Dio acts on GBM cells is to affect the phosphorylation of EGFR, then regulate its downstream signal transduction, and finally trigger the apoptosis process, resulting in the up-regulation of CASP3 expression, a key gene for apoptosis. There may be a relationship between the two mediated by changing the EGFR signaling pathway, which is related to inducing apoptosis, but the specific regulatory network needs to be further studied to elucidate the exact mechanism by which EGFR phosphorylation promotes apoptosis.

Although this study has made some progress, there are still some shortcomings. For example, the theoretical prediction of network pharmacological analysis based on existing data may have limitations of bioinformatics data and imperfect analysis methods. Experimental verification mainly relies on cell experiments and there are still differences with human physiological and pathological environment. Furthermore, while our *in vivo* experiments demonstrated the antitumor efficacy of Dio in a xenograft model, the absence of a positive control group (e.g., TMZ) limits direct comparisons with current standard therapies. We decided to omit a conventional positive control group in the current exploratory phase, as the primary goal was to establish the foundational activity of Dio against GBM and validate its predicted targets (e.g., EGFR/CASP3). Future studies will incorporate TMZ as a positive control to systematically compare the differences in therapeutic efficacy and synergistic potential between Dio and conventional chemotherapeutic agents and aim at perfecting these aspects.

In addition, although the current study does not directly evaluate the BBB penetration of Dio, research on structural analogs of its aglycone derivative, diosgenin (e.g., compound 5b), provides critical insights into Dio’s potential BBB permeability. The research reported that diosgenin, with its smaller molecular weight (414.63 Da) and higher lipophilicity (log*P* ≈ 5.0), can traverse the BBB via passive diffusion and exhibits neuroprotective effects in Alzheimer’s disease models, such as reducing Aβ plaque deposition. Further investigations reveal that structural optimization of diosgenin derivatives (e.g., compound 5b) through the introduction of an indole moiety significantly reduces polar surface area (PSA = 85.118 Å²) and enhances lipophilicity (brain-to-plasma ratio = −0.733), leading to improved cognitive function in murine models [47]. Given that Dio shares a steroidal core structure with diosgenin, its glycosylation modifications may circumvent the limitation of its larger molecular weight (869.06 Da) via active transport mechanisms (e.g., GLUT1-mediated uptake) or nanocarrier-assisted delivery. The physicochemical properties of Dio (e.g., log*P* ≈ 2.5) and structural homology with diosgenin suggest plausible BBB penetration mechanisms. While direct validation through *in vitro* BBB models or *in vivo* pharmacokinetic studies remains necessary, the existing data on diosgenin and its derivatives provide a structurally homologous rationale for hypothesizing Dio’s BBB permeability and establish a theoretical foundation for future research.

Current standard therapies for GBM, including TMZ-based chemotherapy, face significant limitations such as drug resistance, systemic toxicity, and incomplete BBB penetration. TMZ exerts its cytotoxic effects via DNA alkylation at O6-guanine residues; however, resistance frequently arises from overexpression of O6-methylguanine-DNA methyltransferase (MGMT) and enhanced DNA damage repair pathways, such as RAD51-mediated homologous recombination [48]. Recent studies have further elucidated novel resistance mechanisms, including ITPKB-mediated regulation of ROS homeostasis [49] and CENPU/TRIM5α-driven RPS3 ubiquitination, which activates ERK1/2 signaling to promote DNA repair [50]. These pathways collectively underscore the complexity of TMZ resistance and highlight the urgent need for therapeutic strategies targeting non-overlapping molecular mechanisms. In contrast, the natural steroidal saponin Dio, investigated in this study, exhibits multi-target anti-GBM activity. Mechanistically, Dio induces apoptosis by inhibiting EGFR phosphorylation and activating CASP3, independent of DNA damage pathways. This mechanism circumvents MGMT and RAD51-dependent resistance, offering a potential therapeutic strategy for TMZ-resistant tumors. Network pharmacology and experimental validation further demonstrate that Dio dually regulates the EGFR axis and downstream PI3K/AKT-MAPK signaling cascades. Such polypharmacological action starkly contrasts with TMZ’s single-target mechanism and may mitigate resistance by suppressing compensatory signaling pathways (e.g., ERK1/2 activation) [50]. While TMZ resistance is associated with ITPKB-mediated ROS suppression [50], Dio modulates ROS levels through EGFR–CASP3 signaling, potentially reversing pro-survival redox adaptations without exacerbating oxidative stress-related toxicity. Notably, TMZ is limited by dose-dependent myelosuppression and gastrointestinal toxicity. In our subcutaneous GBM xenograft mouse model, intraperitoneal administration of Dio at therapeutic doses (12–24 mg/kg) induced no significant weight loss or behavioral abnormalities, indicating a favorable safety profile. Structural homology between Dio and BBB-penetrant analogs (e.g., diosgenin) suggests its potential to address TMZ’s inadequate cerebrospinal fluid penetration (∼20% of plasma concentration) [48]. Future studies will explore synergistic anti-GBM effects of Dio–TMZ combinations and evaluate its efficacy in recurrent models to advance clinical translation.

In general, this study revealed the relevant core targets and signaling pathways that Dio may participate in the treatment of GBM, and explored at different levels from experiment to network pharmacology, providing an important basis for more in-depth exploration of the pharmacological mechanism of Dio anti-GBM, and expanding the scope of clinical application of Dio in treating GBM.

## 5 Conclusions

Dio, a significant anti-tumor component derived from the seeds of *L. chinensis*, exhibits promising therapeutic effects against GBM. Based on *in vivo* and *in vitro* experimental studies, we found that Dio has a relatively effective anti-tumor effect on GBM. This aroused our interest to further explore its pharmacological mechanism. Then, through network pharmacological analysis, we found that the core target protein of Dio–GBM interaction includes EGFR, which may be closely related to apoptosis signaling pathway. Importantly, the results of network pharmacological analysis were verified by western blotting, and it was found that EGFR phosphorylation and apoptosis gene CASP3 increased with the increase of Dio drug concentration. This may imply that the mechanism by which Dio acts on GBM cells is to affect the phosphorylation of EGFR, then regulate its downstream signal transduction, and finally trigger the apoptosis process, resulting in the up-regulation of CASP3 expression, a key gene for apoptosis. These findings expand the potential clinical application of Dio for GBM.
